# Increasing access to healthful foods: a qualitative study with residents of low-income communities

**DOI:** 10.1186/1479-5868-12-S1-S5

**Published:** 2015-07-27

**Authors:** Alexandra Evans, Karen Banks, Rose Jennings, Eileen Nehme, Cori Nemec, Shreela Sharma, Aliya Hussaini, Amy Yaroch

**Affiliations:** 1 Michael & Susan Dell Center for Healthy Living, The University of Texas School of Public Health Austin Regional Campus, Austin, TX, USA; 2 Share Our Strength Center for Best Practices, 1030 15th St NW, Ste 1100 West, Washington, D.C. 20005, USA; 3 Michael & Susan Dell Foundation, PO Box 163867, Austin, TX 78716, USA; 4 Gretchen Swanson Center for Nutrition, 8401 West Dodge Road, Suite100 Omaha, NE 68114, USA

**Keywords:** qualitative study, access to healthful foods, food insecurity, ethnically diverse, health disparities, focus groups, food access

## Abstract

**Background:**

Inadequate access to healthful foods has been identified as a significant barrier to healthful dietary behaviors among individuals who live in low-income communities. The purpose of this study was to gather low-income community members’ opinions about their food purchasing choices and their perceptions of the most effective ways to increase access to healthful foods in their communities.

**Methods:**

Spanish and English focus groups were conducted in low-income, ethnically-diverse communities. Participants were asked about their knowledge, factors influencing their food purchasing decisions, and their perceptions regarding solutions to increase access to healthful foods.

**Results:**

A total of 148 people participated in 13 focus groups. The majority of participants were female and ethnically diverse (63% Hispanic, 17% African American, 16% Caucasian, and 4% “other”). More than 75% of the participants reported making less than $1999 USD per month. Participants reported high levels of knowledge and preference for healthful foods. The most important barriers influencing healthful shopping behaviors included high price of healthful food, inadequate geographical access to healthful food, poor quality of available healthful food, and lack of overall quality of the proximate retail stores. Suggested solutions to inadequate access included placement of new chain supermarkets in their communities. Strategies implemented in convenience stores were not seen as effective. Farmers’ markets, with specific stipulations, and community gardens were regarded as beneficial supplementary solutions.

**Conclusion:**

The results from the focus groups provide important input from a needs assessment perspective from the community, identify gaps in access, and offer potential effective solutions to provide direction for the future.

## Background

In 2012, food insecurity—or lack of consistent access to enough nutritious food to meet the needs of all household members because of insufficient money or other resources for food—was experienced by approximately 17.6 million households in the United States (U.S.) [1]. Food insecurity places individuals at greater risk for engaging in less healthful dietary behaviors and consuming fewer servings of fruits and vegetables (F&V), dairy foods, and complex micronutrients compared to individuals who are food secure [2,3]. A strong relationship between food insecurity and poverty exists, with higher rates of food insecurity and hunger occurring among individuals of lower socioeconomic status (SES) [2].

Food access is a critical component of food insecurity, and it is often considered a function of a variety of factors, including the spatial proximity to food resources, as well as the affordability, cultural appropriateness, and the nutritional adequacy of available resources. Limited food access has been found to disproportionately affect low-income individuals who are more likely to live in communities with limited availability of healthful foods, specifically fresh fruits and vegetables [1,4-10]. These types of underserved communities, often referred to as “food deserts” [5], tend to have few food retailers who sell healthful food products (e.g., fresh F&V) and more food retailers who sell less healthful foods [11-13]. Low-income individuals living in communities with limited healthful food access tend to have less healthful diets and run a higher risk for chronic disease, such as various cancers, cardiovascular disease, and Type 2 diabetes, compared to individuals living in higher income communities [8,13-15].

According to data provided by the United States Department of Agriculture (USDA), 23.5 million people (approximately 20%) in the U.S. live in low-income communities more than 1 mile from a supermarket. Additional data show that people living in low-income areas with limited access spend significantly more time (19.5 min) traveling to a supermarket compared to the national average (15 min) [16]. As a result of the relatively high prevalence of U.S. households living in communities with limited access and the noted health disparities among those living in these type of communities, federal and local initiatives are underway to increase both geographic and economic access to more healthful foods. Current strategies include increasing *geographic access* by increasing points of healthful food access [17-20]. Although there has been an emphasis on placing more chain supermarkets in food deserts [9], other geographic strategies to improve the healthfulness of the community food environment include changing the inventory of convenience stores (i.e., small retail stores which typically sell a limited variety of staple groceries and snacks), increasing the number of farmers’ markets and farm stands, and establishing community gardens [18,19]. In addition to efforts to increase geographic access to more healthful foods, strategies to increase economic access are also being implemented. These types of strategies include pricing schemes (i.e., decreasing price of more healthful foods and/or increasing price of less healthful foods) at supermarkets, convenience stores, and at farmers’ markets (e.g., *Double Dollar* or *Double Up Food Bucks Program* incentives which provides consumers with incentives that match the value of their federal nutrition benefits when used to purchase fresh, local produce at participating farm-to-retail venues) [21].

Both geographic and economic strategies are being implemented in communities all across the U.S. with relatively little evidence that they are effective and with almost no input from community stakeholders regarding the feasibility and cultural appropriateness of these strategies. The purpose of this paper is to present in-depth qualitative data obtained from focus groups with residents living in underserved, low-income communities about their food purchasing choices and their perceptions of the most effective ways to increase access to more healthful foods in their own communities. The results from the focus groups will provide important input to help inform lay communities as well as identify gaps to help provide direction for future intervention efforts. Although we located two focus groups studies conducted in food deserts in Great Britain [22,23], this study is unique because, to the best of the authors’ knowledge, no other studies in the United States have explored these issues in a qualitative, comprehensive manner.

## Methods

For this study, qualitative data regarding access to more healthful foods (defined in this study as F&V) were collected from 148 adults living in low-income food desert areas in central Texas in spring 2011. Specifically, focus group participants were asked about their knowledge of healthful eating, factors influencing their food purchasing decisions, and their perceptions regarding solutions to increase access to more healthful foods. Institutional Review Board approvals from The University of Texas at Austin and The University of Texas Health Science Center were obtained before commencement of the study.

### Participants

**Figure 1:**
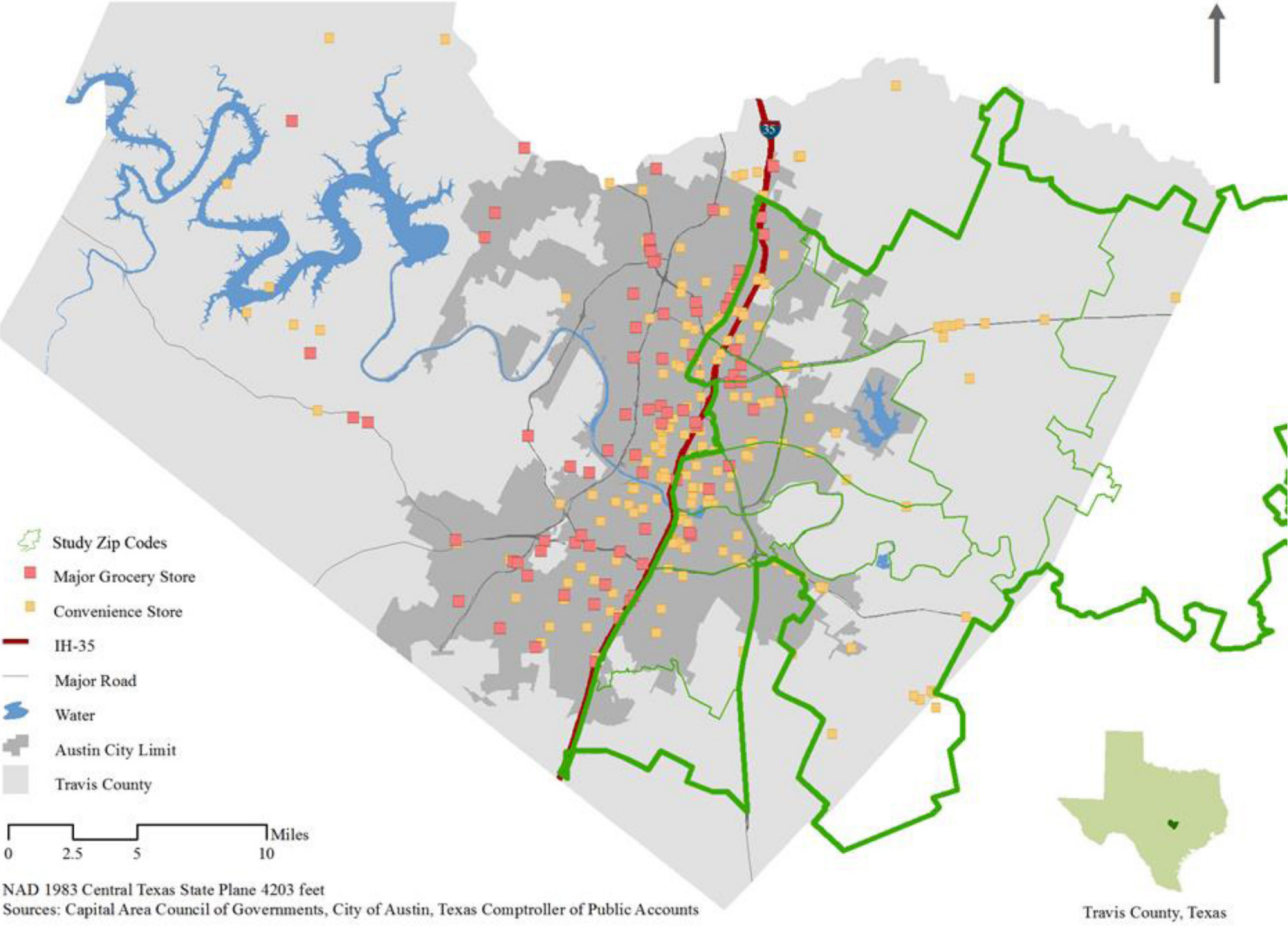
Food stores in study area

Focus group participants were recruited from 11 geographically proximate zip codes, with high concentrations of individuals living in households below the poverty threshold and with limited access to healthy food, as defined by the lack of a chain supermarket in the community within one mile from the majority of residents [24]. Figure 1 illustrates the study area and the location of chain supermarkets within that area. Of the 11 zip codes in the study area, five lacked a supermarket, with the nearest grocery store between 3 and 15 miles away. Table 1 compares demographic information from the study area from which focus group participants were recruited to demographics from Texas and the U.S.

**Table 1:** Comparison of specific demographic variables at study, state and national level

**Demographics**	**Study Area**	**Texas**	**United States**
Median Age	31.3	33.6	37.2
% White	59.9	70.4	72.4
% Black or African American	11.6	11.8	12.6
% Hispanic	49.4	37.2	16.3
% Asian	3.9	3.8	4.8
% Unemployed	9.8	7.3	6.0
Median Income	40,146	50,920	53,046
% Enrolled in SNAP in the Past 12 Months	19.17	11.2	11.4
% Individuals Below Poverty Level	25.75	17	10.9
% High School Graduates	71.78	80.8	85.7
% Spanish Speaking Household	46.71	29.3	29.4
% Living in Same House a Year Ago	77.85	82.1	n/a

^1^Source: U.S. Census Bureau, 2010 Census

^2^Source: U.S. Census Bureau, 2008-2012 American Community Survey

^3^ Poverty is determined when the total household income of the householder’s family falls below the appropriate poverty threshold (determined by: size of family, number of related children, and, for 1- and 2-person families, age of householder).

^4^ Average percentage graduation rates of all 11 zip codes represented by study participants.

In order to recruit a random sample of community residents, over 20 community leaders, including church pastors, social service providers, non-profit directors, and neighborhood association members, were contacted to help determine venues and times for the focus groups. Flyers were distributed to schools, churches, community recreation centers, select businesses, and door-to-door. Inclusion criteria for participation were: 1) resident of one of the 11 zip codes included in our study, 2) responsibility for purchase of most household food, and 3) ages 18-65 years. While this study explicitly focused on the needs of low-income residents, specific income level was not a requirement for participation. However, given that the communities were all considered low income, it was assumed that most of the participants would be low income and exposed to a low-income community environment. Interested and eligible individuals were asked to contact the research team or attend a scheduled focus group meeting. For participating in the focus groups, participants were given a small bag of local farm-fresh produce worth approximately $20 USD.

### Focus group

The focus group sessions were interactive discussions guided by 15 open-ended questions using a standardized focus group protocol that lasted approximately one hour [25]. The questions were developed using the U.S. Department of Agriculture (USDA) concept of *food security* as a guiding framework. The USDA defines *food security* as *access by all people at all times to enough nutritious food for an active, healthy life* and encompasses both geographic and economic access to healthy food [1]. Questions were developed to specifically examine participants’ perceptions with regard to what constitutes a more healthful diet, factors influencing food-purchasing decisions, and how to increase geographic and economic access to healthful food in the participants’ communities. Three examples of questions included in this study were: 1) How often do you normally purchase food? 2) What are some reasons that limit the amount of fresh fruits and vegetables you buy? 3) What would help increase your consumption of fresh fruits and vegetables? Each question had specific prompts that were used when necessary. The questions focused on three specific venues where residents are able to obtain food: supermarkets, convenience stores, and farmers’ markets. These venues were chosen because they provide, in theory, equal access to all community members living in a specific community. During the focus groups, participants also mentioned community and school gardens, thus these points of access will also be discussed in this paper. The focus group scripts were developed in English, then translated into Spanish, and then back translated into English by a native-Spanish speaker.

### Data collection

A total of 13 focus groups (7= Spanish; 6=English) were conducted (n=148) by trained moderators with experience in conducting both English and Spanish focus groups. In addition to the regular moderator, an assistant moderator was used for each focus group as well. Spanish focus groups were conducted either by a trained, fluent Spanish speaker or by a trained English speaker accompanied by a bilingual translator. Before the start of each session, research staff obtained written informed consent from all participants. All study materials were available in both English and Spanish. At the start of the focus groups, participants completed a short socio-demographic (e.g., race/ethnicity, sex, age, participant employment, and food security status) and specific food behaviors survey (e.g., frequency of food preparation, frequency of dinner eaten at home). The socio-demographic questions were drawn from an earlier study conducted with a very similar population [26]. The sessions lasted approximately 45-60 minutes and all sessions were audio taped. Upon completion of the focus groups, trained research assistants transcribed the audiotapes. Spanish focus groups were transcribed in Spanish, checked for accuracy against original recordings, translated into English by a native Spanish speaker, and back-translated into Spanish for quality control.

### Data analysis

In order to describe the sample, frequencies for specific variables on the quantitative questionnaire were calculated. For the qualitative data analysis, a thematic content analysis approach was used. Each transcript was entered into the qualitative software package QSR NVivo (version 8, 2008, QSR International Pty Ltd, Cambridge, MA). Two independent coders experienced in the analysis of qualitative data reviewed the interviews and decided on a coding scheme based on the focus group questions and the recurrent themes found in the transcripts. In addition, a set of decision rules to standardize the coding procedure was created. The two coders then went back through all the transcripts and assigned specific codes to each segment of text that corresponded to each index code and corresponding subcategories. Organization of coded and sub-coded passages of the transcribed text was examined and differences in coding were resolved through consensus by the two coders. Emergent themes were identified through frequency of coding within similar contexts and across focus groups. All index codes that were mentioned less than three times were not included in further analyses. [25].

## Results and discussion

### Participants

A total of 148 people participated in 13 focus groups. The majority of participants were female and the total sample was ethnically diverse: 63% Hispanic, 17% African American, 16% Caucasian, and 4% “other.” Among Hispanic participants, the majority (54%) reported speaking Spanish most of the time. Approximately three-fourths of participants (72%) reported making less than $1999 USD per month and 68% reported *sometimes* or *almost always* “running out of food by the end of the month.” (See Table 2).

**Table 2:** Description of focus group participants (n = 148)

	**Number**	**Percent**
Gender		
Female	107	85%
Male	41	15%
Ethnicity/Race		
Black or African American	25	16.89%
Hispanic or Latino	94	63.51%
White	24	16.22%
Other	5	3.38%
Language spoken at home		
Spanish	80	54.05%
English	65	43.92%
Other	2	1.35%
I don’t know	1	0.68%
Marital Status		
Married	72	48.65%
Separated or divorced	30	20.27%
Single, never married	34	22.97%
Widowed	12	8.11%
Highest Level of Education		
Less than 12 years	44	29.73%
High school graduate/GED	47	31.76%
Some college	23	15.54%
College graduate	27	18.24%
Missing	7	6.08%
Total Household Income/Month		
$0 – 999 USD	55	37.16%
$1000 – 1999	51	34.46%
$2000 – 2999	14	9.46%
$3000 – 3999	3	2.03%
$4000 +	13	8.78%
Missing	12	9.46%
Receive WIC vouchers		
No	117	79.05%
Yes	31	20.95%
Receive Supplemental Nutrition Assistance Benefits (SNAP)		
No	99	66.89%
Yes	43	29.05%
Missing	6	5.41%
Run out of food before end of month because can’t afford to buy more		
Almost always	55	37.16%
Sometimes	45	30.41%
Not very often	48	32.43%

According to the Center for Public Priorities, a family of four with two adults needs to earn a gross monthly income between $4198 USD to afford to “get by” in Central Texas [27]. Based on this estimate, only about 12% percent of the focus group participants earned enough to afford to live comfortably in Central Texas. However, only 30% of the participants received SNAP benefits and only 21% received Women, Infants, and Children (WIC) benefits, which may be one reason 68% of the participants reported *sometimes* or *almost always* feeling food insecure. These numbers suggest that strategies that increase economic access to food are important for this population.

### Summary of qualitative results

The following themes were derived from the data: 1) High level of knowledge about healthy eating, 2) Factors influencing food purchasing decisions include: cost of food (economic access), distance to retail store (geographic access), quality of food and quality of retail stores, 3) Suggested ways to improve physical access to healthful foods through supermarkets, convenience stores, farmers’ markets and community gardens, 4) Suggested way to increase economic access to healthful foods.

### Knowledge of healthful eating

A review of psychosocial factors associated with fruit and vegetables intake among adults showed knowledge as a significant predictor of F&V consumption across an array of socio-demographic populations [28]. In our study, focus group participants were very knowledgeable of what it means to eat healthy. The majority of the participants used consumption of fruits and vegetables (F&V) as a proxy when answering questions about healthy foods in general. Participants unanimously agreed that a variety of F&V is an essential part of a more healthful diet. They listed F&V as more healthful because—in their opinion—these foods provide vitamins, nourishment, strength, help lower cholesterol, cause one to think clearly, and prevent diet-related diseases. On participant noted that F&V “help your body balance and process everything properly.” The words “fresh,” “organic,” “seasonal” and “local” were all mentioned in connection to F&V and health, in that respective order of frequency. Results from other studies concur with our findings that levels of knowledge about healthy foods among low-income shoppers tends to be high [29], suggesting that lack of knowledge is not the driving factor influencing food purchasing and dietary behaviors among this population [26,30,31].

### Factors influencing food purchasing decisions

Despite the high level of knowledge about the components of a healthy diet, participants voiced several external barriers to consuming more healthful foods. The four most common influences reported included high cost of healthful foods, inadequate geographical access to healthful food, poor quality of available healthful food, and lack of overall quality of the proximate retail stores. Focus group participants identified high cost as the number one factor affecting food choice: “*We always look for what’s more economical*.” For families with limited financial resources, the need to stay within a fixed budget caused a trade-off between more healthful foods and, oftentimes, less healthy but calorie-dense foods, such as meat. As one participant reported: “*I look at the asparagus and I realize that I can buy a big rib eye for the same price so I get the rib eye*.” F&V were viewed as very healthy but not as satiating as other foods, posing a dilemma for families who were forced to choose between their health values and meeting their basic caloric needs. The price of food and budget restrictions also limited families’ options in the variety of foods purchased and ways food was prepared. Participants reported “*rarely*” or “*not regularly*” shopping with a prepared grocery list, rather shopping for the same products every week or “*looking for special sales*” because the cost of the food and preparation methods were known and the amount of food wasted was limited. Similar results were found in another focus group study with residents of food deserts. In this study, younger mothers with children cited financial constraints as greatly influencing their food purchasing decisions [22].

Although cost of food was the dominating factor affecting food-purchasing decisions, the distance to a supermarket or large grocery store that carried higher quality products was also a major concern for participants. In some cases, residents reported having to travel up to 20 miles to buy groceries. Particularly for participants who did not own a car, transportation to supermarkets was a hardship and potentially very expensive as some participants reported taking a taxi to the store due to poor and uncoordinated public transportation. Even participants who did own a car cited high gasoline prices as a barrier to driving to supermarkets outside their community. As one participant noted, “*I always look for the closest place because I can save gas and sometimes there are things that are cheaper at certain places, and we know they are on sale, but also if the store is too far you have to have in mind the traffic, the time and the gas, so I prefer to buy the food in the one that is closest whether it’s more expensive or not*.” For many of the families in the study, grocery shopping is not a solitary errand. It requires forethought to incorporate this activity into one’s daily commute or combine with other errands in order to save gas money and requires advanced preparation (e.g., placing a cooler full of ice in the car so food does not spoil).

Quality of both the available foods and the retail stores were also consistent factors mentioned as important influences on food purchasing decisions. Terms like *fresh, not mildewed, not wilted, not bruised, not rotten, good appearance, good shape*, and *pretty* were used to describe food of high quality. High quality stores were described as having a *nice physical condition*, *clean, good upkeep, not too much traffic or panhandling in the parking lot*. Many participants who did live near a supermarket or grocery store mentioned that the quality of foods, especially produce and prepared foods, at the local supermarkets and grocery stores was greatly inferior compared to food items sold at other supermarkets across town in higher-income neighborhoods on the west side of town (Figure 1). In fact, some participants stated that when they had the opportunity, they would try to go to a store much further away because the quality of foods found at those stores was better. However, among other participants this was rare, since it was hard to justify a trip to a better store with the high price of gas “*because if it’s too expensive, it’s not convenient for you to go too far because you’ll spend more [on gas] than what you have*.” Thus, even though these individuals had relatively easy geographic access to a supermarket, they did not have access to *quality* food. This underscores the need to not only provide geographic and economic access, but also access to quality products.

In summary, results from the focus groups confirmed that both economic and geographic access are major factors influencing how low-income individuals shop for their food. The four specific factors that influence how and where food purchases are made include: price of food, geographic access, quality of food for sale, and quality of store. Other studies have found similar results and underscore the importance of the affordability, variety, and quality of food as well as proximity to grocery stores as main influences on where to shop [31-36].

### Increasing geographic access to healthful foods

When asked how access to healthful foods could be improved, responses depended somewhat on the geographic location of where participants lived. However, all focus group conversations included comments about supermarkets, convenience stores, and alternative venues such as farmers’ markets and community gardens.

#### Supermarkets

Participants living in areas with no supermarket consistently stated that the solution to increase access to more healthful foods was to build a conveniently located supermarket offering a wide variety of quality items in a convenient location. For those participants already living close to a supermarket, solutions focused on increasing the quality of food available in the store and on improving the overall quality of the store. Some participants also mentioned expanding services provided by stores and have stores create common spaces for community classes on how to grow food and hosting regular farmers’ markets.

Participants specifically preferred supermarkets over smaller-sized grocery stores because supermarkets offered a variety of other needed services (e.g., payment of bills, etc.). One participant shared her thoughts about a supermarket: “*Whenever you go to [the store], you can also pay your bills; it’s faster, you can use other services there. That way, I only go to one place and at the same time I get my groceries like the fruits, the tuna, the nopales [cactus], everything is there, ready!*” For some participants, a larger size meant variety: “*I like the big [store] because it’s got everything in there. I mean you could just go in there and have a field day. You can shop!*” This result was also found in a focus group conducted in an area of Great Britain considered to be a food desert [22].

Given that the most commonly cited barriers to the purchase of more healthful foods by participants were cost, lack of geographic access, and lack of quality produce, introducing new supermarkets in communities would seem to be a logical solution. However, past studies indicate that the simple placement of a new supermarket in a food desert or similar type of community does not necessarily translate into an increase of healthier food purchasing or healthier food intake [37-41]. Only two studies have found significant positive results after a new grocery store was introduced into a community [37,41]. The results from one study show that among participants with “very poor” diets at pre-intervention, F&V consumption increased from 4.13 portions to 9.83 portions per week, and among participants with “poor” diets, 60% increased F&V consumption [37]. It is important to note that while each of the studies examined the impact of a new supermarket, there is a lack of consistency across each of the studies. For example, while the new supermarket evaluated by Sadler et al. (2013) increased geographic access, the authors imply that food prices were relatively higher at this independent grocer compared to other available alternatives; thus, in this low-income community, economic access was not improved by the new store [38]. One reason for the mixed results may be that supermarkets increase availability of *both* healthful and unhealthful foods, which may translate into more purchases of both healthful and unhealthful foods [42].

On the other hand, results from studies assessing the impact of intervention strategies placed *within an already existing supermarket*, including increasing availability of healthful foods and making healthful foods more affordable, tend to be positive. A review of 58 articles published from the late 1940s to July 2012 evaluating the impact of interventions implemented in supermarkets to promote healthful food choices and eating practices found that the combination of pricing, increasing availability of healthful foods, points-of-purchase signage, and advertising is an effective strategy to increase the purchase of more healthful food items [43]. A recent study by Waterlander et al. (2013) also found that a 50% discount on fresh F&V throughout a six-month period in supermarkets resulted in a significant increase in F&V purchases and consumption [44]. At a three month follow-up, after price discounts had ceased, the impact on F&V purchasing and consumption had ceased as well, suggesting that price adjustments must be maintained to maintain purchasing behaviors, especially since F&V tend to be more price elastic than other foods [45].

In summary, evidence from the literature suggests that increasing geographic access by simply placing supermarkets in food deserts may not increase the purchase and consumption of healthful foods. However, altering costs of foods and increasing availability of healthful foods in already-existing stores does seem to positively impact consumers’ purchasing and consumption behaviors. Given that placing a grocery store in a low-income area can be a lengthy and complicated process (e.g., lengthy approval procedures from city government) and is not always an economically practical option for retailers [46], improving the quality of already-existing grocery stores, if available, may be a more viable option. If no supermarket or grocery store is already available, then introducing a new supermarket that offers competitive prices and a variety of affordable, high quality foods will increase access and potentially will increase more healthful dietary behaviors, including increased F&V consumption.

#### Convenience stores

When participants were specifically asked about their use of convenience stores for food purchasing, the overall sentiment was very negative. Convenience stores were typically perceived to be too expensive, as reflected by a participant who stated, “*…I’d prefer to grab my car and go to Store A (local chain retail store) instead. It’s more economical*.” Another participant felt that convenient stores “*conveniently make that price ridiculous*.” Convenience stores also were perceived as having limited and very low-quality food products, especially produce. One participant was put out by having to go to corner stores since there weren’t enough large grocery stores nearby, noting “*most of them have processed foods*.” Participants reported a general feeling of frustration and mistrust towards convenience store businesses. One participant expressed, “*My thing is that I don’t [shop at] the convenience store, even though I’m wasting 5 or 6 bucks worth of gas not going in, I’m still not going to give him $5 or $6 for a pack of bacon. I can’t do it. I would rather spend the $5 or $6 on gas and go to [store B]. Them knowing there is no access to this type of stuff so they mark the food up real high. That’s not cool*.”

Transformation of convenience stores to sell healthier foods has been posited as an alternative or interim solution to the introduction of chain supermarkets. Gittelsohn et al. (2012) published a review of 16 original articles examining the impact of strategies to increase access to more healthful foods in convenience stores [47]. The most common strategies tested in the reviewed studies focused on increasing the availability of healthier foods, reducing the availability of unhealthy foods, reducing the cost of healthy, foods or providing cash incentives for healthful foods. Results of the review found that food purchasing and consumption patterns improved in 9 of the 10 trials that assessed this outcome [47]. In addition, results from a more recent study showed that greater exposure to the intervention (i.e., increasing healthy food availability in local food stores and promotion of these foods through point-of-purchase and community media interventions) was associated with significantly reduced body mass index (p≤0.05) and improved healthy food intentions (p≤0.01), healthy cooking methods (p≤0.05) and healthy food purchasing (p≤0.01) [48]. Another recent study assessed the effectiveness of an initiative to increase the availability and promotion of healthier food options in 55 convenience stores in New York City. The percentage of consumers surveyed who purchased healthier options that were promoted through the initiative increased from 5% to 16% [49]. In summary, evidence from the literature suggests that implementing strategies that increase the availability and affordability of healthful foods in convenience stores can be effective in increasing healthier food purchasing and consumption.

Although published studies have shown positive outcomes of strategies tested within convenience stores, participants of our focus group study perceived convenience stores as offering lower quality foods and preferred to go without or drive the extra distance to access larger stores that offered more variety at a more reasonable cost. Our focus group results differed from another study conducted with mostly African-American participants in New Orleans which showed that this population was highly likely to shop at convenience stores [50]. These mixed results could be due to the perception that—in our study—store owners lacked community investment because they were from outside the community. It is also possible that car ownership is greater in Texas compared to other states. Having a car gives one the ability to forego an undesirable store for one that is further away [51]. However, since gas was a big expense for our participants, owning a car may not necessarily solve the issue of lack of a proximal supermarket.

Given the negative perception of convenience stores by members of our focus groups, efforts to incentivize convenience stores to carry a larger variety of healthier products may not be the most effective solution in communities similar to the communities where our focus group study was conducted. In order for our participants to shop at convenience stores, negative perceptions about convenience store businesses need to be improved first. If perceptions are not changed, individuals may not perceive conveniences stores as a viable option for the purchase of foods and thus will not be affected by the strategies implemented within the convenience stores.

#### Farmers’ markets

Farmer’s markets as a point of access for purchasing fresh produce were discussed among participants with varied reactions. Conceptually, farmers’ markets were appealing, with the provision of easily accessible, fresh, and often organic produce. However, there was also resistance to farmers’ markets based on reactions to previous experiences with existing farmers’ markets. A majority of the participants reported that current markets were too expensive, too far away, and operated at inconvenient times. Although many of the participants had heard about farmers’ markets, only a few were familiar with their current locations, which was not convenient for the majority of the focus group participants. Produce at farmers markets was seen as high quality but also as cost-prohibitive. One community member remarked, “*I’ve been and I liked it because the veggies taste different, the tomatoes taste different, everything is fresh…but it’s more expensive*.”

Farmers’ markets were also critiqued for the undesirable quantities of produce and lack of other goods compared to grocery stores: “*There is not a lot of variety, I have been but there is not too much variety, just like tomatoes and peppers*.” Additionally, the preset quantities of produce offered at some farmers’ markets were either too large or too small for what participants needed. One person remarked, “*The thing is that they [farmers’ markets] already have the quantity that they want to sell. Like how they have the tomatoes in a little basket…sometimes you can’t buy the tomatoes because they are $3, and so you don’t have enough money left to buy the peppers. I can’t just buy what I need… On the other hand, if you go to [store B] you can just buy one tomato*.”

Past studies indicate that introducing new farmers’ markets or farm stands in low income communities can increase F&V intake among the residents who live close to the farmers’ market [52]. Additional studies have also examined the impact of farmers’ market-based economic strategies on nutrition-related outcomes. McCormack et al (2012) reviewed 12 studies which all focused on providing economic incentives for specific populations to purchase F&V at farmers’ markets [53]. In general, the results from these studies suggest that providing economic incentives for purchasing produce at farmers’ markets increases both purchasing of produce at farmers’ markets and vegetable intake among participants [53]. Another study by Freedman et al (2013) examined the effect of placing farmers’ markets at federally qualified health clinics along with offering financial incentives to low-income diabetics and found a marginally significant increase in F&V consumption [54]. Thus, in summary, farmers’ markets may serve as an alternative venue to offer fresh, high quality produce. When combined with financial schemes such as the *Double Dollar* program or the *Farmers’ Market Nutrition Program* (FMNP)—an incentive program which allows recipients of the Special Supplemental Nutrition Program for Women, Infants, and Children (WIC) to purchase locally grown produce sold at farmers’ markets with free FMNP coupons—the dollars spent at the farmers’ market can be stretched and more produce can be bought at a lower price [55].

Evidence from both the published literature and the focus groups in the current study suggest that adding more farmers’ markets can be a positive improvement for fresh and high quality F&V access, as long at the markets operate at consistent and convenient times and at convenient locations. Convenient locations suggested by participants for new farmers’ markets included local schools and parking lots of grocery stores. Adding strategies to increase economic access to markets may be especially effective.

#### Community gardens

Although not a direct point of access for *food purchasing*, gardening and community gardens were brought up by focus group participants as alternative ways to improve access to fresh produce. The notion of growing one’s food was tangible, and to some participants represented a waning generational skill: “*… like we did many years ago, you know, right out of the garden. Right there from your farm, your own animals, your own vegetables too*.” However, the issue of lack of time was often connected to the idea of gardening to supplement healthy foods: “*Growing your own veggies, I have always wanted to do that but I never have time*.” At the same time, gardening carried with it other intangible benefits, especially for their children, that seemed to outweigh the challenge of time: “*I think it’s important…it’s important to find a way to have the time to teach kids how to cultivate plants and take care of them, love them, and then eat them…it would be a different and new culture in the kids’ lives*.” Parents in many of the focus groups favored the idea of school gardens as a viable option for increasing food access and teaching children about healthy foods, and several parents shared stories about the impact of existing school gardens had on their children.

A recent review article measuring the nutritional effects of community gardens found that community garden participants reported a higher likelihood of F&V intake compared to non-gardeners [53]. A more recent longitudinal study reported the results of an intervention designed to support Hispanic farmworker families to grow a home garden. The intervention provided home garden materials, group educational and social activities, and volunteer assistance to participating families. Results indicated that at the end of the study, participants reported increased vegetable intake compared to baseline measurement [56].

Results from the published literature and our focus group findings found positive results for the use of community gardens on individuals’ F&V consumption. Although community gardens can rarely feed entire communities, these results suggest that community gardens and possibly school gardens can serve as way to both educate and create higher demand for locally grown produce. Thus, additional efforts in creating more of these points of access and providing additional education on how to grow produce could be relatively easy and inexpensive strategies to implement in low-income communities.

### Increasing economic access to healthful foods

Although general food prices have tended to decrease over the past decades, food prices for F&V increased by 17% between 1997 and 2003 [57]. Given that higher priced F&V are associated with child BMI [58], decreasing the cost of these specific healthful foods is critical. When asked for potential solutions to the high cost of more healthful food, participants provided several strategies that they currently use to help with food costs. Many participants reported often seeking out sales or specials and comparing store prices in order to be able to purchase more for less. One participant referred to herself and her friends as “coupon-aholics” in order to save money. Similar findings were reported in a study with low-income individuals who identified strategies such as using coupons, limiting variety, gardening, purchasing dented cans, and diluting as ways to stretch a food dollar [59].

Another way that participants save money is to prepare meals for their families at home. Responses from the focus groups and to the survey questions indicated that most participants consistently prepared at least one meal per day, mainly dinner, at home. Data from the short quantitative questionnaire that participants completed before the focus groups indicated that over 52% of respondents reported that their family eats dinner together at home almost every day while another 31% eat together more than 50% of the time (data not shown). Eating at home was reported to be both more economical and healthier. Lastly, in order to save money on produce, some respondents reported buying produce in season only.

### Limitations and strengths

This study utilized a convenience sample and included only adults living in low-income communities with limited access to healthful foods. This limits the generalizability of this study to the geographic region in which the study was conducted. However, because the purpose of this study was to specifically obtain more in-depth information from this particular population, our inclusion criteria were fairly specific. Because the focus groups were not conducted according to ethnic/racial/language groups, it is not possible to discuss any cultural, language, or ethnic/racial differences. One strength of this qualitative study is the large number of participants, which increases the generalizability of the findings, especially within Texas. Future studies should be designed to allow for the examination of cultural and ethnic/racial differences among the focus group participants. In addition, examining differences according to access to transportation are needed as well.

## Conclusions

Findings from this study suggest that solutions to the issue of inadequate access to more healthful foods need to be multifaceted and approached using a system-level framework, especially because the economic and the geographic constraints tend to be inextricably linked. The first step is to ensure geographic access to retail stores that provide more healthful, higher quality and, most importantly, affordable food. Affordability of more healthful foods can be addressed both by reducing their price and through incentives such as Double Dollars/Double Up Food Bucks. In addition to providing healthful foods, the retail store itself needs to be physically attractive, clean and safe. In-store marketing of healthful foods and health promotion and education can also increase accessibility. Farmers’ markets and community gardens can serve as less expensive complementary solutions and can serve to increase demand for more locally-grown and fresh produce. A higher demand for fresh produce can benefit local farmers, a traditionally low-income population group, as well. In addition, increased demand will possibly increase willingness of larger retailers to place new stores in the community, which ultimately can bring both economic and health benefits to the community.

## Competing interests

The authors declare that they do not have competing interests.

## Authors’ contributions

AE conceptualized the manuscript, conducted the analysis, and prepared the first draft of the manuscript. KB, RJ, and EN participated in conceptualizing the analyses, in examining the results of analysis, and in drafting the paper. KB led the data collection and was assisted by RJ and AE. SS, AH, and AY all provided critical review of multiple drafts. Collection of data and the analytical / writing effort on this work were made possible by funding from the Sustainable Food Center, as well as a grant from the Michael & Susan Dell Foundation.

## Declarations

This article has been published as part of *International Journal of Behavioral Nutrition and Physical Activity* Volume 12 Supplement 1, 2015: The science of childhood obesity: an individual to societal framework. The full contents of the supplement are available online at http://www.ijbnpa.org/supplements/12/S1. Publication costs for this supplement were funded by the Michael & Susan Dell Foundation.
